# Supplementary material for: Deferred Administration of Afobazole Induces Sigma1R-Dependent Restoration of Striatal Dopamine Content in a Mouse Model of Parkinson’s Disease
Source: Int J Mol Sci. 2020 Oct 15;21(20):7620. doi: 10.3390/ijms21207620 (PMC7593947; doi:10.3390/ijms21207620)
Supplement: Supplementary file 1 [file ijms-21-07620-s001.docx]

Supplementary Table 1. The influence of Sigma1R ligands on content of dopamine and its metabolites in striata of sham-operated and 6-OHDA-lesioned mice.

| **Experimental groups** |  |  | **DA**  **nmole/g tissue** | **DOPAC**  **nmole/g tissue** | **HVA**  **nmole/g tissue** |
| --- | --- | --- | --- | --- | --- |
| Vehicle | Sham | C  n = 10 | 84.04 ± 6.61 | 5.21 ± 0.62 | 13.47 ± 1.85 |
|  |  | I  n = 10 | 88.26 ± 9.5 | 5.3 1± 0.49 | 13.54 ± 1.5 |
|  |  | C  n = 11 | 87.95 ± 10.81 | 5.02 ± 0.54 | 13.79 ± 2.12 |
|  |  | I  n = 12 | 36.84 ± 18.86  + p < 0.0001  * p < 0.0001 | 2.71 ± 1.43  + p < 0.0001  * p < 0.0001 | 7.12 ± 3.63  + p < 0.0001  * p < 0.0001 |
| BD-1047 | Sham | C  n = 6 | 82.54 ±1 4.73 | 5.9 6± 1.04 | 11.56 ± 1.52 |
|  |  | I  n = 6 | 78.7 ± 11.68 | 5.82 ± 1.14 | 11.84 ± 2.29 |
|  | 6-OHDA | C  n = 10 | 89.9 ± 12.46 | 6.49 ± 1.34 | 12.98 ± 3.33 |
|  |  | I  n = 9 | 51.82 ± 24.35  + p = 0.0007  * p < 0.0001  ^ p = 0.0099 | 3.46 ± 1.65  + p = 0.0001  * p = 0.0025  ^ p = 0.0006 | 7.96 ± 3.47  + p = 0.003  * p < 0.0001  ^ p = 0.044 |
| Afobazole | Sham | C  n = 6 | 79.45 ± 4.41 | 5.07 ± 0.8 | 11.96 ± 0.93 |
|  |  | I  n = 6 | 78.02 ± 4.43 | 5.1 ± 0.7 | 10.97 ± 1.0 |
|  | 6-OHDA | C  n = 12 | 97.45 ± 8.88 | 5.76 ± 0.64 | 14.99 ± 2.54 |
|  |  | I  n = 12 | 82.7 ± 13.7  + p = 0.0008  # p < 0.0001 | 5.42 ± 0.94  # p < 0.0001 | 13.89 ± 2.28  + p = 0.02  # p < 0.0001 |
| BD-1047 + Afobazole | Sham | C  n = 6 | 79.81 ± 8.84 | 6.69 ± 1.1 | 13.37 ± 3.05 |
|  |  | I  n = 6 | 87.48 ± 4.62 | 6.03 ± 0.93 | 13.53 ± 1.67 |
|  | 6-OHDA | C  n = 11 | 91.81 ± 10.28 | 6.01 ± 0.57 | 14.18 ± 1.74 |
|  |  | I  n = 11 | 54.51 ± 13.46  + p < 0.0001  * p < 0.0001  ^ p = 0.0002  *a* p < 0.0001 | 3.46 ± 0.5  + p < 0.0001  * p = 0.0062  ^ p = 0.0003  *a* p = 0.0031 | 8.83 ± 1.34  + p < 0.0001  * p = 0.0001  ^ p = 0.0026  *a* p < 0.0001 |
| PRE-084 | Sham | C  n = 6 | 77.62 ± 2.43 | 5.46 ± 0.73 | 12.38 ± 0.9 |
|  |  | I  n = 6 | 76.8 ± 1.91 | 5.64 ± 0.31 | 12.54 ± 0.72 |
|  | 6-OHDA | C  n = 9 | 88.49 ± 9.95 | 5.87 ± 0.92 | 12.31 ± 1.79 |
|  |  | I  n=9 | 76.14 ± 7.79  + p = 0.0012  # p < 0.0001 | 5.69 ± 1.67  # p < 0.0001 | 11.57 ± 1.04  # p < 0.0001 |
| BD-1047 + PRE-084 | Sham | C  n=6 | 79.3 ± 7.87 | 6.59 ± 1.04 | 12.99 ± 1.84 |
|  |  | I  n=6 | 79.93 ± 5.47 | 6.23 ± 1.0 | 13.35 ± 1.41 |
|  | 6-OHDA | C  n=11 | 89.86 ± 9.66 | 5.99 ± 0.98 | 13.62 ± 1.26 |
|  |  | I  n=11 | 52.13 ± 12.36  + p < 0.0001  * p < 0.0001  ^ p = 0.004  *p* p = 0.0049 | 3.46 ± 0.51  + p < 0.0001  * p = 0.0062  ^ p < 0.0001  *p* p = 0.0005 | 8.89 ± 1.39  + p < 0.0001  * p = 0.0001  ^ p = 0.0055  *p* p = 0.028 |

Data are presented as mean ± S.D. Sham – sham-operated animals. 6-OHDA – animals with 6-OHDA lesion. C – contralateral striatum. I – ipsilateral striatum. Paired t-test: + - statistical significance vs. contralateral striatum. Two-way ANOVA, Tukey multiple comparison test statistical significance: * vs. sham-operated vehicle-treated mice. # 6-OHDA-lesioned vehicle-treated mice. ^ vs. respective group of sham-operated animals. *a* vs. 6-OHDA-lesioned afobazole-treated mice. *p* vs. 6-OHDA-lesioned PRE-084-treated mice.

Supplementary Table 2. Influence of Sigma1R ligands on dopamine turnover in striata of sham-operated and 6-OHDA-lesioned ICR mice

|  |  |  |  |  |  |
| --- | --- | --- | --- | --- | --- |

| **Experimental groups** | | | **DOPAC/DA** | **HVA/DA** | **(DOPAC+HVA)/DA** |
| --- | --- | --- | --- | --- | --- |
| Vehicle | Sham | C  n = 10 | 0.062 ± 0.005 | 0.16±0.012 | 0.22±0.014 |
|  |  | I  n = 10 | 0.061 ± 0.006 | 0.16±0.015 | 0.22±0.018 |
|  | 6-OHDA | C  n = 11 | 0.058 ± 0.007 | 0.16±0.032 | 0.22±0.036 |
|  |  | I  n = 12 | 0.083 ± 0.03  + p = 0.027 | 0.23±0.13 | 0.32±0.16  + p=0.042 |
| BD-1047 | Sham | C  n = 6 | 0.075 ± 0.021 | 0.14±0.023 | 0.22±0.049 |
|  |  | I  n = 6 | 0.074 ± 0.008 | 0.15±0.019 | 0.22±0.022 |
|  | 6-OHDA | C  n = 10 | 0.072 ± 0.001 | 0.15±0.037 | 0.22±0.043 |
|  |  | I  n = 9 | 0.093 ± 0.069 | 0.21±0.17 | 0.31±0.24 |
| Afobazole | Sham | C  n = 6 | 0.064 ± 0.007) | 0.15±0.014 | 0.21±0.013 |
|  |  | I  n = 6 | 0.065 ± 0.007 | 0.14±0.014 | 0.21±0.013 |
|  | 6-OHDA | C  n = 12 | 0.059 ± 0.005 | 0.15±0.019) | 0.21±0.022 |
|  |  | I  n = 12 | 0.065 ± 0.012 | 0.17±0.02  + p=0.019 | 0.23±0.026  + p=0.021 |
| BD-1047 + Afobazole | Sham | C  n = 6 | 0.086 ± 0.025 | 0.17±0.062 | 0.26±0.086 |
|  |  | I  n = 6 | 0.069 ± 0.01 | 0.15±0.016 | 0.22±0.024 |
|  | 6-OHDA | C  n = 11 | 0.066 ± 0.009 | 0.17±0.028 | 0.22±0.024 |
|  |  | I  n = 11 | 0.067±0.017 | 0.15±0.019 | 0.23±0.044 |
| PRE-084 | Sham | C  n = 6 | 0.08±0.0097 | 0.17±0.015 | 0.24±0.014 |
|  |  | I  n = 6 | 0.075±0.006 | 0.17±0.0095 | 0.24±0.01 |
|  | 6-OHDA | C  n = 9 | 0.067±0.011 | 0.14±0.011 | 0.21±0.017 |
|  |  | I  n = 9 | 0.075 ± 0.022 | 0.15 ± 0.023  + p = 0.043 | 0.23 ± 0.032  + p=0.0096 |
| BD-1047 + PRE-084 | Sham | C  n = 6 | 0.083 ± 0.011 | 0.17 ± 0.033 | 0.25 ± 0.04 |
|  |  | I  n = 6 | 0.078 ± 0.0097 | 0.17 ± 0.015 | 0.24 ± 0.014 |
|  | 6-OHDA | C  n = 11 | 0.066 ± 0.007 | 0.15 ± 0.025 | 0.22 ± 0.026 |
|  |  | I  n = 11 | 0.068 ± 0.012 | 0.18 ± 0.037 | 0.25 ± 0.045 |

|  |  |  |  |  |  |
| --- | --- | --- | --- | --- | --- |

Data are presented as mean ± S.D. Sham – sham-operated animals. 6-OHDA – animals with 6-OHDA lesion. C – contralateral striatum. I – ipsilateral striatum. Paired t-test: + - statistical significance vs. contralateral striatum.

Supplementary Table 3. The influence of Sigma1R ligands on rotarod performance of sham-operated and 6-OHDA-lesioned ICR mice.

| **Experimental groups** | | **FSRR, s** | **ARR, s** |
| --- | --- | --- | --- |
| Vehicle | Sham  n = 10 | 119.5  (109.0 - 120.0) | 180.0  (163.8-180.0) |
|  | 6-OHDA  n = 12 | 27.5  (18.3-36.0)  * p < 0.001 | 73.5  (53.3 - 90.0)  * p < 0.001 |
| BD-1047 | Sham  n = 6 | 119.0  (108.5 - 120.0) | 180.0  (161.3 - 180.0) |
|  | 6-OHDA  n = 10 | 34.0  (25.5 - 61.5)  * p = 0.002  ^ p = 0.016 | 71.0  (53.5 - 85.5)  * p < 0.001  ^ p = 0.006 |
| Afobazole | Sham  n = 6 | 117.5  (112.3-120.0) | 180.0  (170.5 - 180.0) |
|  | 6-OHDA  n = 12 | 85.5  (70.5 - 115.5)  # p = 0.022 | 167.0  (150.8 - 174.8)  # p = 0.015 |
| BD-1047 + Afobazole | Sham  n = 6 | 120.0  (116.5 - 120.0) | 180.0  (178.3 - 180.0) |
|  | 6-OHDA  n = 11 | 29.5  (15.75-44.75)  * p < 0.001  *a* p = 0.027  ^ p < 0.001 | 78.0  (63.0 - 88.0)  * p = 0.001  *a* p = 0.048  ^ p = 0.001 |
| PRE-084 | Sham  n = 6 | 120.0  (107.0-120.0) | 180.0  (173.5 - 180.0) |
|  | 6-OHDA  n = 9 | 92.0  (74.5 - 115.5)  # p = 0.025 | 165.0  (153.0 - 170.0)  # p = 0.046 |
| BD-1047 + PRE-084 | Sham  n = 6 | 119.5  (111.5 - 120.0) | 178.5  (167.3 - 180.0) |
|  | 6-OHDA  n = 11 | 26.0  (16.0 - 46.0)  * p < 0.001  *p* p = 0.049  ^ p = 0.003 | 67.0  (37.0 - 82.0)  * p < 0.001  *p* p = 0.028  ^ p < 0.002 |

Data are presented as median (q25 - 75). Sham – sham-operated animals. 6-OHDA – animals with 6-OHDA lesions. n – the number of animals in the experimental group. Kruskal‒Wallis test, Dunn’s multiple comparison test: * - statistical significance vs. sham-operated vehicle-treated mice; # - statistical significance vs. 6-OHDA-lesioned vehicle-treated mice; ^ - statistical significance vs. corresponding sham-operated group; *a* – statistical significance vs. afobazole-treated mice with 6-OHDA lesions; *p* – statistical significance vs. PRE-084-treated mice with 6-OHDA lesions.
